# Supplementary material for: The Characteristics of Patients That Develop Severe Leptospirosis: A Scoping Review
Source: Pathogens. 2025 Dec 10;14(12):1268. doi: 10.3390/pathogens14121268 (PMC12735429; doi:10.3390/pathogens14121268)
Supplement: Supplementary file 1 [file pathogens-14-01268-s001.zip › pathogens-4011651-supplementary.pdf]

**Supplementary file S1** Database Search strategy

Search performed on 2/3/2025

| Database | Combined |
|---|---|
| Pubmed | (("Cohort"[Title/Abstract] OR ("case"[Title/Abstract] AND "control"[Title/Abstract] AND "series"[Title/Abstract]) OR ("case"[Title/Abstract] AND "control"[Title/Abstract]) OR "Retrospective"[Title/Abstract] OR "Prospective"[Title/Abstract] OR "Observational"[Title/Abstract] OR ("Cross"[Title/Abstract] AND "sectional"[Title/Abstract]) OR ("clinical"[Title/Abstract] AND "audit"[Title/Abstract]) OR (case-control studies[MeSH Terms]) OR (cohort studies[MeSH Terms]) OR (Clinical audit[MeSH Terms]) OR (Prospective Studies[MeSH Terms]) OR (Observational Studies[MeSH Terms]) OR (Cross-Sectional Studies[MeSH Terms]) OR (Retrospective Studies[MeSH Terms])) AND ("Severities"[Title/Abstract] OR "Severity"[Title/Abstract] OR "sever*"[Title/Abstract] OR "Prognosis"[Title/Abstract] OR "Predictive"[Title/Abstract] OR "Prediction"[Title/Abstract] OR "Prognosis"[MeSH Terms] OR "Severity of Illness Index"[MeSH Terms] OR "Severity of Illness Index"[MeSH Terms]) AND (((((("Leptospira"[Title/Abstract] AND "Infection*"[Title/Abstract]) OR ((weil*[Title/Abstract] OR swineherd[Title/Abstract] OR stuttgart[Title/Abstract]) AND (disease*[Title/Abstract]))) OR ((mud[Title/Abstract] OR rice[Title/Abstract] OR cane[Title/Abstract] OR canicola[Title/Abstract]) AND (fever[Title/Abstract]))) OR ("spirochetal"[Title/Abstract] AND "Jaundice"[Title/Abstract])) OR (Leptospiros*[Title/Abstract] OR Leptospirosis[Title/Abstract] OR Leptospiroses[Title/Abstract])) OR ((Leptospirosis[MeSH Terms]) OR (Weil Disease[MeSH Terms]))) |
| Scopus | (((((((TITLE-ABS(Leptospira) AND TITLE-ABS(Infection*)) OR ((TITLE-ABS(weil*) OR TITLE-ABS(swineherd) OR TITLE-ABS(stuttgart)) AND (TITLE-ABS(disease*)))) OR ((TITLE-ABS(mud) OR TITLE-ABS(rice) OR TITLE-ABS(cane) OR TITLE-ABS(canicola)) AND (TITLE-ABS(fever)))) OR (TITLE-ABS(spirochetal) AND TITLE-ABS(Jaundice))) OR (TITLE-ABS(Leptospiros*) OR TITLE-ABS(Leptospirosis) OR TITLE-ABS(Leptospiroses))) OR ((INDEXTERMS(Leptospirosis)) OR (INDEXTERMS("Weil Disease")))) AND (TITLE-ABS(Severities) OR TITLE-ABS(Severity) OR TITLE-ABS(sever*) OR TITLE-ABS(Prognosis) OR TITLE-ABS(Predictive) OR INDEXTERMS(prediction) OR INDEXTERMS(Prognosis) OR INDEXTERMS("Severity of Illness Index") OR INDEXTERMS("Severity of Illness Index"))) AND ((TITLE-ABS(Cohort) OR (TITLE-ABS(case) AND TITLE-ABS(control) AND TITLE-ABS(series)) OR (TITLE-ABS(case) AND TITLE-ABS(control)) OR TITLE-ABS(Retrospective) OR TITLE-ABS(Prospective) OR TITLE-ABS(Observational) OR (TITLE-ABS(Cross) AND TITLE-ABS(sectional)) OR (TITLE-ABS(clinical) AND TITLE-ABS(audit))) OR (INDEXTERMS("“Cohort Studies”")) OR (INDEXTERMS("“case-control studies”")) OR (INDEXTERMS("“cohort studies”")) OR (INDEXTERMS("“Clinical audit”")) OR (INDEXTERMS("“Prospective Studies”")) OR (INDEXTERMS("“Observational Study”")) OR (INDEXTERMS("“Cross-Sectional Studies”")) OR (INDEXTERMS("“Retrospective Studies”"))) |
| Medline | (((((((Leptospira.tw. AND Infection*.tw.) OR ((weil*.tw. OR swineherd.tw. OR stuttgart.tw.) AND (disease*.tw.))) OR ((mud.tw. OR rice.tw. OR cane.tw. OR canicola.tw.) AND (fever.tw.))) OR (spirochetal.tw. AND Jaundice.tw.)) OR (Leptospiros*.tw. OR Leptospirosis.tw. OR Leptospiroses.tw.)) OR ((exp Leptospirosis/) OR (exp "Weil Disease"/))) AND (Severities.tw. OR Severity.tw. OR sever*.tw. OR Prognosis.tw. OR prediction.tw. OR Predictive.tw. OR exp Prognosis/ OR exp "Severity of Illness Index"/ OR exp "Severity of Illness Index"/)) AND ((Cohort.tw. OR (case.tw. AND control.tw. AND series.tw.) OR (case.tw. AND control.tw.) OR Retrospective.tw. OR Prospective.tw. OR Observational.tw. OR (Cross.tw. AND sectional.tw.) OR (clinical.tw. AND audit.tw.)) OR (exp “Cohort Studies”/) OR (Exp “case-control studies”/) OR (exp “cohort studies”/) OR (Exp “Clinical audit”/) OR (Exp “Prospective Studies”/) OR (exp “Observational Study”/) OR (exp “Cross-Sectional Studies”/) OR (Exp “Retrospective Studies”/)) |
| Emcare | (((((((Leptospira.tw. AND Infection*.tw.) OR ((weil*.tw. OR swineherd.tw. OR stuttgart.tw.) AND (disease*.tw.))) OR ((mud.tw. OR rice.tw. OR cane.tw. OR canicola.tw.) AND (fever.tw.))) OR (spirochetal.tw. AND Jaundice.tw.)) OR (Leptospiros*.tw. OR Leptospirosis.tw. OR Leptospiroses.tw.)) OR ((exp Leptospirosis/) OR (exp "Weil Disease"/))) AND (Severities.tw. OR Severity.tw. OR sever*.tw. OR Prognosis.tw. OR Predictive.tw. OR exp Prognosis/ OR prediction.tw. OR exp "disease severity"/ OR exp "Severity of Illness Index"/ OR exp “disease severity assessment”/ OR exp “predictive model”/)) AND (Cohort.tw. OR (case.tw. AND control.tw. AND series.tw.) OR (case.tw. AND control.tw.) OR Retrospective.tw. OR Prospective.tw. OR Observational.tw. OR (Cross.tw. AND sectional.tw.) OR (clinical.tw. AND audit.tw.) OR ((exp “Cohort Study”/) OR (exp “case-control study”/) OR (exp “cohort study”/) OR (exp “Clinical audit”/) OR (exp “Prospective Study”/) OR (exp “Observational Study”/) OR (exp “Cross-Sectional Study”/) OR (exp “Retrospective Study”/))) |
| CINAH | ((((((((TI Leptospira OR AB Leptospira) AND (TI Infection* OR AB Infection*)) OR (((TI weil* OR AB weil*) OR (TI swineherd OR AB swineherd) OR (TI stuttgart OR AB stuttgart)) AND ((TI disease* OR AB disease*)))) OR (((TI mud OR AB mud) OR (TI rice OR AB rice) OR (TI cane OR AB cane) OR (TI canicola OR AB canicola)) AND ((TI fever OR AB fever)))) OR ((TI spirochetal OR AB spirochetal) AND (TI Jaundice OR AB Jaundice))) OR ((TI Leptospiros* OR AB Leptospiros*) OR (TI Leptospirosis OR AB Leptospirosis) OR (TI Leptospiroses OR AB Leptospiroses))) OR (((MH Leptospirosis+)) OR ((MH "Weil Disease+")))) AND ((TI Severities OR AB Severities) OR (TI Severity OR AB Severity) OR (TI sever* OR AB sever*) OR (TI Prognosis OR AB Prognosis) OR (TI Predictive OR AB Predictive) OR (MH Prognosis+) OR (MH "Severity of Illness Index+") OR (MH "Severity of Illness Index+"))) AND (((TI Cohort OR AB Cohort) OR ((TI case OR AB case) AND (TI control OR AB control) AND (TI series OR AB series)) OR ((TI case OR AB case) AND (TI control OR AB control)) OR (TI Retrospective OR AB Retrospective) OR (TI Prospective OR AB Prospective) OR (TI Observational OR AB Observational) OR ((TI Cross OR AB Cross) AND (TI sectional OR AB sectional)) OR ((TI clinical OR AB clinical) AND (TI audit OR AB audit))) OR ((MH "“Cohort Studies”+")) OR ((MH "“case-control studies”+")) OR ((MH "“cohort studies”+")) OR ((MH "“Clinical audit”+")) OR ((MH "“Prospective Studies”+")) OR ((MH "“Observational Study”+")) OR ((MH "“Cross-Sectional Studies”+")) OR ((MH "“Retrospective Studies”+"))) |

**Supplementary file S2.** Preferred Reporting Items for Systematic Reviews and Meta-Analysis (PRISMA)

| SECTION | ITEM | PRISMA-ScR CHECKLIST ITEM | REPORTED ON PAGE # |
|---|---|---|---|
| **TITLE** | | | |
| Title | 1 | Identify the report as a scoping review. | 1 |
| **ABSTRACT** | | | |
| Structured summary | 2 | Provide a structured summary that includes (as applicable): background, objectives, eligibility criteria, sources of evidence, charting methods, results, and conclusions that relate to the review questions and objectives. | 1 |
| **INTRODUCTION** | | | |
| Rationale | 3 | Describe the rationale for the review in the context of what is already known. Explain why the review questions/objectives lend themselves to a scoping review approach. | 2 |
| Objectives | 4 | Provide an explicit statement of the questions and objectives being addressed with reference to their key elements (e.g., population or participants, concepts, and context) or other relevant key elements used to conceptualize the review questions and/or objectives. | 2 |
| **METHODS** | | | |
| Protocol and registration | 5 | Indicate whether a review protocol exists; state if and where it can be accessed (e.g., a Web address); and if available, provide registration information, including the registration number. | 3 |
| Eligibility criteria | 6 | Specify characteristics of the sources of evidence used as eligibility criteria (e.g., years considered, language, and publication status), and provide a rationale. | 3, Appendix 1 |
| Information sources* | 7 | Describe all information sources in the search (e.g., databases with dates of coverage and contact with authors to identify additional sources), as well as the date the most recent search was executed. | 3, Appendix 1 |
| Search | 8 | Present the full electronic search strategy for at least 1 database, including any limits used, such that it could be repeated. | Appendix 1 |
| Selection of sources of evidence† | 9 | State the process for selecting sources of evidence (i.e., screening and eligibility) included in the scoping review. | 3, Figure 1 |
| Data charting process‡ | 10 | Describe the methods of charting data from the included sources of evidence (e.g., calibrated forms or forms that have been tested by the team before their use, and whether data charting was done independently or in duplicate) and any processes for obtaining and confirming data from investigators. | 3 |
| Data items | 11 | List and define all variables for which data were sought and any assumptions and simplifications made. | 3 |

| SECTION | ITEM | PRISMA-ScR CHECKLIST ITEM | REPORTED ON PAGE # |
|---|---|---|---|
| Critical appraisal of individual sources of evidence§ | 12 | If done, provide a rationale for conducting a critical appraisal of included sources of evidence; describe the methods used and how this information was used in any data synthesis (if appropriate). | 3-4, Appendix 3 |
| Synthesis of results | 13 | Describe the methods of handling and summarizing the data that were charted. | 3 |
| **RESULTS** | | | |
| Selection of sources of evidence | 14 | Give numbers of sources of evidence screened, assessed for eligibility, and included in the review, with reasons for exclusions at each stage, ideally using a flow diagram. | 2-4, Figure 1 |
| Characteristics of sources of evidence | 15 | For each source of evidence, present characteristics for which data were charted and provide the citations. | Table 1 |
| Critical appraisal within sources of evidence | 16 | If done, present data on critical appraisal of included sources of evidence (see item 12). | 3-4, Appendix 3 |
| Results of individual sources of evidence | 17 | For each included source of evidence, present the relevant data that were charted that relate to the review questions and objectives. | Table 1 |
| Synthesis of results | 18 | Summarize and/or present the charting results as they relate to the review questions and objectives. | 13-17 |
| **DISCUSSION** | | | |
| Summary of evidence | 19 | Summarize the main results (including an overview of concepts, themes, and types of evidence available), link to the review questions and objectives, and consider the relevance to key groups. | 18-20 |
| Limitations | 20 | Discuss the limitations of the scoping review process. | 19-20 |
| Conclusions | 21 | Provide a general interpretation of the results with respect to the review questions and objectives, as well as potential implications and/or next steps. | 20 |
| **FUNDING** | | | |
| Funding | 22 | Describe sources of funding for the included sources of evidence, as well as sources of funding for the scoping review. Describe the role of the funders of the scoping review. | 21 |

JBI = Joanna Briggs Institute; PRISMA-ScR = Preferred Reporting Items for Systematic reviews and Meta-Analyses extension for Scoping Reviews.
* Where *sources of evidence* (see second footnote) are compiled from, such as bibliographic databases, social media platforms, and Web sites.
† A more inclusive/heterogeneous term used to account for the different types of evidence or data sources (e.g., quantitative and/or qualitative research, expert opinion, and policy documents) that may be eligible in a scoping review as opposed to only studies. This is not to be confused with *information sources* (see first footnote).
‡ The frameworks by Arksey and O'Malley (6) and Levac and colleagues (7) and the JBI guidance (4, 5) refer to the process of data extraction in a scoping review as data charting.
§ The process of systematically examining research evidence to assess its validity, results, and relevance before using it to inform a decision. This term is used for items 12 and 19 instead of "risk of bias" (which is more applicable to systematic reviews of interventions) to include and acknowledge the various sources of evidence that may be used in a scoping review (e.g., quantitative and/or qualitative research, expert opinion, and policy document).

**Supplementary file S3.** Risk of bias assessment using Newcastle-Ottawa score

| Study | Selection | Comparability | Exposure | Overall |
|---|---|---|---|---|
| Suwannarong *et al*. 2014 [1] | + | − | + | + |
| Al Hariri *et al*. 2022 [2] | + | + | + | + |
| Pongpan *et al*. 2023 [3] | + | + | + | + |
| Sandhu *et al*. 2020 [4] | + | − | + | − |
| Rajapakse *et al*. 2015 [5] | + | − | + | + |
| Lee *et al*. 2017 [6] | + | + | + | + |
| Panaphut *et al*. 2002 [7] | + | − | + | + |
| Goswami *et al*. 2014 [8] | + | − | + | + |
| Li *et al*. 2022 [9] | + | − | + | + |
| Fonseka *et al*. 2023 [10] | + | − | + | + |
| Philip *et al*. 2021 [11] | − | + | + | + |
| Nisansala *et al*. 2023 [12] | + | − | + | + |
| Wang *et al*. 2020 [13] | + | − | + | + |
| Budiono *et al*. 2009 [14] | + | − | + | + |
| Ajjimarungsi *et al*. 2020 [15] | + | − | + | + |
| Silva *et al*. 2024 [16] | + | − | + | + |
| Daher Ede *et al*. 2019 [17] | + | + | + | + |
| Spichler *et al*. 2008 [18] | + | − | + | + |
| Galdino *et al*. 2023 [19] | + | + | + | + |
| Daher Ede *et al*. 2016 [20] | + | + | + | + |
| Marotto *et al*. 2010 [21] | + | + | + | + |

| | | | | |
|---|---|---|---|---|
| Herrmann-Storck *et al*. 2010 [22] | + | − | + | + |
| Hochedez *et al*. 2015 [23] | + | − | + | + |
| Sharp *et al*. 2016 [24] | − | + | + | − |
| Dupont *et al*. 1997 [25] | + | − | + | + |
| Miailhe *et al*. 2019 [26] | + | × | + | − |
| Delmas *et al*. 2018 [27] | + | − | + | + |
| Petakh *et al*. 2022 [28] | − | − | + | − |
| Gancheva 2016 [29] | + | − | + | + |
| Esen *et al*. 2004 [30] | + | + | + | + |
| Abgueguen *et al*. 2008 [31] | + | − | + | + |
| Smith *et al*. 2019 [32] | + | − | + | + |
| Craig *et al*. 2009 [33] | + | × | + | + |
| Tubiana *et al*. 2013 [34] | + | − | + | + |
| Mikulski *et al*. 2014 [35] | + | − | + | + |

| Judgement: |
|---|
| × High |
| − Some concerns |
| + Low |

*Case Controls and Cohort studies*

| Domains: | Domain scoring: High | Domain scoring: some concerns | Domain scoring: Low |
|---|---|---|---|
| D1: Bias due to selection | 0-1 | 2 | 3+ |
| D2: Bias due to comparability | 0 | 1 | 2+ |
| D3: Bias due to outcome | 0 | 1 | 2+ |
| Overall | 0-3 | 4-6 | 7-9 |

Cross sectional studies

| Domains: | Domain scoring: High | Domain scoring: Some concerns | Domain scoring: Low |
|---|---|---|---|
| D1: Bias due to selection | 0 | 1 | 2+ |
| D2: Bias due to comparability | 0 | 1 | 2+ |
| D3: Bias due to outcome | 0 | 1 | 2+ |
| Overall | 0-2 | 3-5 | 6-7 |

**References**


1. Suwannarong K, Singhasivanon P, Chapman RS. Risk factors for severe leptospirosis of Khon Kaen Province: A case-control study. *Journal of Health Research*. 2014;28(1):59
EP - 64.
2. Al Hariri YK, Sulaiman SAS, Khan AH, Adnan AS, Al-Ebrahem SQ. Determinants of prolonged hospitalization and mortality among leptospirosis patients attending tertiary care hospitals in northeastern state in peninsular Malaysia: A cross sectional retrospective analysis. *Front Med (Lausanne)*. 2022;9:887292. doi:https://dx.doi.org/10.3389/fmed.2022.887292
3. Pongpan S, Thanatrakolsri P, Vittaporn S, Khamnuan P, Daraswang P. Prognostic Factors for Leptospirosis Infection Severity. *Trop Med Infect Dis*. Feb 11 2023;8(2)doi:10.3390/tropicalmed8020112
4. Sandhu RS, Ismail HB, Ja'afar MHB, Rampal S. The Predictive Factors for Severe Leptospirosis Cases in Kedah. *Trop Med Infect Dis*. May 14 2020;5(2)doi:10.3390/tropicalmed5020079
5. Rajapakse S, Weeratunga P, Niloofa MJ, et al. Clinical and laboratory associations of severity in a Sri Lankan cohort of patients with serologically confirmed leptospirosis: a prospective study. *Trans R Soc Trop Med Hyg*. Nov 2015;109(11):710-6. doi:10.1093/trstmh/trv079
6. Lee N, Kitashoji E, Koizumi N, et al. Building prognostic models for adverse outcomes in a prospective cohort of hospitalised patients with acute leptospirosis infection in the Philippines. *Transactions of the Royal Society of Tropical Medicine and Hygiene*. 2017;111(12):531
EP - 539. doi:https://dx.doi.org/10.1093/trstmh/try015
7. Panaphut T, Domrongkitchaiporn S, Thinkamrop B. Prognostic factors of death in leptospirosis: a prospective cohort study in Khon Kaen, Thailand. *Int J Infect Dis*. Mar 2002;6(1):52-9. doi:10.1016/s1201-9712(02)90137-2
8. Goswami RP, Goswami RP, Basu A, Tripathi SK, Chakrabarti S, Chattopadhyay I. Predictors of mortality in leptospirosis: An observational study from two hospitals in Kolkata, eastern India. Article. *Transactions of the Royal Society of Tropical Medicine and Hygiene*. 2014;108(12):791-796. doi:10.1093/trstmh/tru144
9. Li D, Liang H, Yi R, et al. Clinical characteristics and prognosis of patient with leptospirosis: A multicenter retrospective analysis in south of China. *Frontiers in Cellular and Infection Microbiology*. 2022;12:1014530. doi:https://dx.doi.org/10.3389/fcimb.2022.1014530
10. Fonseka CL, Dahanayake NJ, Mihiran DJD, et al. Pulmonary haemorrhage as a frequent cause of death among patients with severe complicated Leptospirosis in Southern Sri Lanka. *PLoS Negl Trop Dis*. Oct 2023;17(10):e0011352. doi:10.1371/journal.pntd.0011352
11. Philip N, Lung Than LT, Shah AM, Yuhana MY, Sekawi Z, Neela VK. Predictors of severe leptospirosis: a multicentre observational study from Central Malaysia. *BMC Infectious Diseases*. 2021;21(1):1-6. doi:10.1186/s12879-021-06766-5
12. Nisansala GGT, Weerasekera M, Ranasinghe N, et al. Predictors of severe leptospirosis on admission: a Sri Lankan study. *International Journal of Infectious*

*Diseases*. 2023/08/01/ 2023;134:S18-S19. doi:https://doi.org/10.1016/j.ijid.2023.05.067
13. Wang HK, Lee MH, Chen YC, Hsueh PR, Chang SC. Factors associated with severity and mortality in patients with confirmed leptospirosis at a regional hospital in northern Taiwan. *J Microbiol Immunol Infect*. Apr 2020;53(2):307-314. doi:10.1016/j.jmii.2018.05.005
14. Budiono E, Riyanto BS, Hisyam B, Hartopo AB. Pulmonary involvement predicts mortality in severe leptospirosis patients. Article. *Acta medica Indonesiana*. 2009;41(1):11-14.
15. Ajjimarungsi A, Bhurayanontachai R, Chusri S. Clinical characteristics, outcomes, and predictors of leptospirosis in patients admitted to the medical intensive care unit: A retrospective analysis. *J Infect Public Health*. Dec 2020;13(12):2055-2061. doi:https://dx.doi.org/10.1016/j.jiph.2020.10.004
16. Silva AFD, Figueiredo K, Falcao IWS, Costa FAR, da Rocha Seruffo MC, de Moraes CCG. Study of machine learning techniques for outcome assessment of leptospirosis patients. *Scientific Reports*. 2024;14(1):13929. doi:https://dx.doi.org/10.1038/s41598-024-62254-1
17. Daher EF, Soares DS, Galdino GS, et al. Leptospirosis in the elderly: the role of age as a predictor of poor outcomes in hospitalized patients. *Pathog Glob Health*. May 2019;113(3):117-123. doi:10.1080/20477724.2019.1621729
18. Spichler AS, Vilaça PJ, Athanazio DA, et al. Predictors of lethality in severe leptospirosis in urban Brazil. *Am J Trop Med Hyg*. Dec 2008;79(6):911-4.
19. Galdino GS, de Sandes-Freitas TV, de Andrade LGM, et al. Development and validation of a simple machine learning tool to predict mortality in leptospirosis. *Scientific Reports*. 2023;13(1):4506. doi:https://dx.doi.org/10.1038/s41598-023-31707-4
20. Daher Ede F, Soares DS, de Menezes Fernandes AT, et al. Risk factors for intensive care unit admission in patients with severe leptospirosis: a comparative study according to patients' severity. *BMC Infect Dis*. Feb 1 2016;16:40. doi:10.1186/s12879-016-1349-x
21. Marotto PC, Ko AI, Murta-Nascimento C, et al. Early identification of leptospirosis-associated pulmonary hemorrhage syndrome by use of a validated prediction model. *J Infect*. Mar 2010;60(3):218-23. doi:10.1016/j.jinf.2009.12.005
22. Herrmann-Storck C, Saint-Louis M, Foucand T, et al. Severe leptospirosis in hospitalized patients, Guadeloupe. *Emerg Infect Dis*. Feb 2010;16(2):331-4. doi:10.3201/eid1602.090139
23. Hochedez P, Theodose R, Olive C, et al. Factors Associated with Severe Leptospirosis, Martinique, 2010-2013. *Emerg Infect Dis*. Dec 2015;21(12):2221-4. doi:10.3201/eid2112.141099
24. Sharp TM, Rivera García B, Pérez-Padilla J, et al. Early Indicators of Fatal Leptospirosis during the 2010 Epidemic in Puerto Rico. *PLoS Negl Trop Dis*. Feb 2016;10(2):e0004482. doi:10.1371/journal.pntd.0004482
25. Dupont H, Dupont-Perdrizet D, Perie JL, Zehner-Hansen S, Jarrige B, Daijardin JB. Leptospirosis: prognostic factors associated with mortality. *Clin Infect Dis*. Sep 1997;25(3):720-4. doi:10.1086/513767

26. Miailhe A-F, Mercier E, Maamar A, et al. Severe leptospirosis in non-tropical areas: a nationwide, multicentre, retrospective study in French ICUs. *Intensive Care Medicine*. 2019;45(12):1763-1773. doi:10.1007/s00134-019-05808-6
27. Delmas B, Jabot J, Chanareille P, et al. Leptospirosis in ICU: A Retrospective Study of 134 Consecutive Admissions. *Critical Care Medicine*. 2018;46(1):93-99. doi:10.1097/CCM.0000000000002825
28. Petakh P, Isevych V, Griga V, Kamyshnyi A. The risk factors of severe leptospirosis in the Transcarpathian region of Ukraine – search for „red flags". Article. *Archives of the Balkan Medical Union*. 2022;57(3):231-237. doi:10.31688/ABMU.2022.57.3.02
29. Gancheva G. Age as prognostic factor in leptospirosis. *Ann Infect Dis Epidemiol 2016; 1 (2)*. 2016;1006
30. Esen S, Sunbul M, Leblebicioglu H, Eroglu C, Turan D. Impact of clinical and laboratory findings on prognosis in leptospirosis. *Swiss Med Wkly*. Jun 12 2004;134(23-24):347-52. doi:10.4414/smw.2004.10436
31. Abgueguen P, Delbos V, Blanvillain J, et al. Clinical aspects and prognostic factors of leptospirosis in adults. Retrospective study in France. *Journal of Infection*. Sep 2008;57(3):171-8. doi:https://dx.doi.org/10.1016/j.jinf.2008.06.010
32. Smith S, Kennedy BJ, Dermedgoglou A, et al. A simple score to predict severe leptospirosis. *PLoS Negl Trop Dis*. Feb 2019;13(2):e0007205. doi:10.1371/journal.pntd.0007205
33. Craig SB, Graham GC, Burns MA, Dohnt MF, Smythe LD, McKay DB. Haematological and clinical-chemistry markers in patients presenting with leptospirosis: a comparison of the findings from uncomplicated cases with those seen in the severe disease. *Ann Trop Med Parasitol*. Jun 2009;103(4):333-41. doi:10.1179/136485909x435058
34. Tubiana S, Mikulski M, Becam J, et al. Risk factors and predictors of severe leptospirosis in New Caledonia. *PLoS Negl Trop Dis*. 2013;7(1):e1991. doi:10.1371/journal.pntd.0001991
35. Mikulski M, Boisier P, Lacassin F, et al. Severity markers in severe leptospirosis: a cohort study. *Eur J Clin Microbiol Infect Dis*. Apr 2015;34(4):687-95. doi:https://dx.doi.org/10.1007/s10096-014-2275-8
